# Abolished ketamine effects on the spontaneous excitatory postsynaptic current of medial prefrontal cortex neurons in GluN2D knockout mice

**DOI:** 10.1186/s13041-021-00883-7

**Published:** 2021-12-07

**Authors:** Dae Hee Han, Ilgang Hong, Ja Eun Choi, Pojeong Park, Jun-Yeong Baek, HyoJin Park, Soichiro Ide, Masayoshi Mishina, Kazutaka Ikeda, Bong-Kiun Kaang

**Affiliations:** 1 School of Biological Sciences, Seoul National University, 1, Gwanak-ro, Gwanak-gu, Seoul, 08826 Korea; 2 Addictive Substance Project, Tokyo Metropolitan Institute of Medical Science, 2- 1-6 Kamikitazawa, Setagaya-ku, Tokyo, 156-8506 Japan; 3 Brain Science Laboratory, The Research Organization of Science and Technology, Ritsumeikan University, 1-1-1 Nojihigashi, Kusatsu, Shiga 525-8577 Japan

**Keywords:** Ketamine, Medial prefrontal cortex, *N*-methyl-d-aspartate receptor (NMDAR), Spontaneous excitatory postsynaptic current (sEPSC), GluN2D

## Abstract

Ketamine, a non-competitive antagonist of the *N*-methyl-d-aspartate receptor (NMDAR), generates a rapidly-acting antidepressant effect. It exerts psychomimetic effects, yet demands a further investigation of its mechanism. Previous research showed that ketamine did no longer promote hyperlocomotion in GluN2D knockout (KO) mice, which is a subunit of NMDAR. In the present study, we tested whether GluN2D-containing NMDARs participate in the physiological changes in the medial prefrontal cortex (mPFC) triggered by ketamine. Sub-anesthetic dose of ketamine (25 mg/kg) elevated the frequency of spontaneous excitatory postsynaptic currents (sEPSC) in wild-type (WT) mice, but not in GluN2D KO mice, 1 h after the injection. The amplitude of sEPSC and paired-pulse ratio (PPR) were unaltered by ketamine in both WT and GluN2D KO mice. These findings suggest that GluN2D-containing NMDARs might play a role in the ketamine-mediated changes in glutamatergic neurons in mPFC and, presumably, in ketamine-induced hyperlocomotion.

Ketamine is an *N*-methyl-d-aspartate receptor (NMDAR) antagonist and has been widely used as an anesthetic drug over the past two decades. Due to its rapid antidepressant effect, ketamine became a breakthrough in the clinical research of depression. Ketamine exerts a rapid and long-lasting antidepressant effect in a dose-dependent manner [1], where a sub-anesthetic dose of ketamine (0.3–1 mg/kg in humans [2] and 5–10 mg/kg in animals [3], respectively) has been reported to be effective in alleviating depressive symptoms [2]. However, ketamine also has notable side effects, such as psychotomimetic symptoms, abuse potential, and neurotoxicity. For instance, a higher dose of ketamine (25–50 mg/kg) triggered dissociation [4] and hyperlocomotion in mice [5].

The NMDAR subunit family is composed of GluN1, GluN2A-D, and GluN3A-B subunits. The GluN2D-containing NMDARs reach maximal expression at the first postnatal week and become restricted in a few cell types including interneurons of the hippocampus and the prefrontal cortex [6, 7]. Previous research has implicated GluN2D-containing NMDARs in the sustained antidepressant effect [8] and the cognitive impairment effect [9] of (*R*)-ketamine, an enantiomer of racemic ketamine. Furthermore, GluN2D knockout (KO) mice did not develop ketamine-induced locomotor sensitization [5]. However, the physiological mechanism through which GluN2D-containing NMDARs contribute to ketamine-induced hyperlocomotion remains largely unknown. To address this question, we measured spontaneous excitatory postsynaptic currents (sEPSC) and paired-pulse ratio in the medial prefrontal cortex (mPFC) layer 5 pyramidal neurons of wild-type (WT) and GluN2D KO mice 1 h after the injection of a sub-anesthetic dose of ketamine, a dose known to trigger hyperlocomotion in rodents (Fig. 1A).

**Fig. 1:**
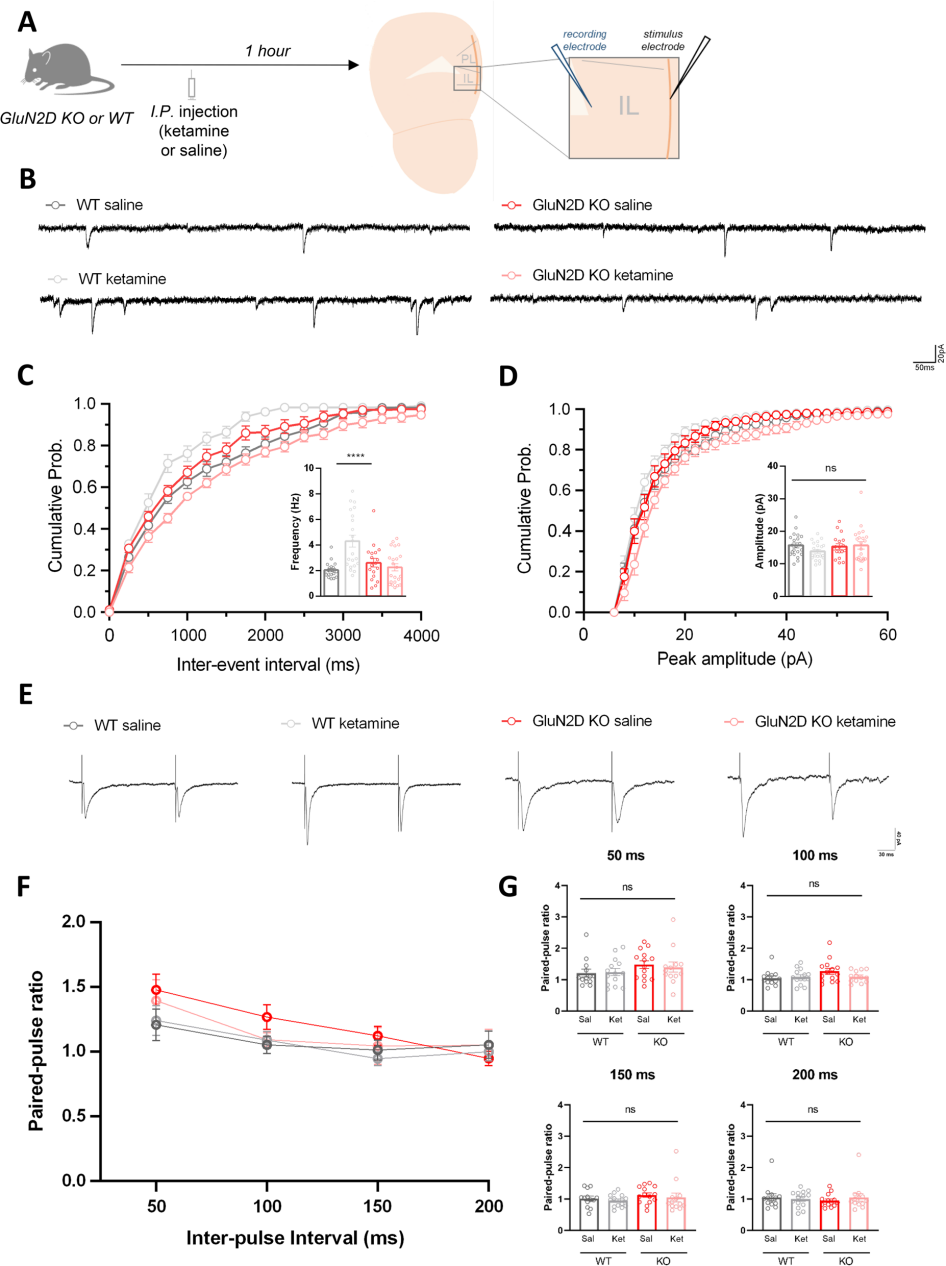
Effects of ketamine on the sEPSC of mPFC neurons. (**A**) Experimental scheme and the recording site in the mPFC. (**B**) Representative traces of sEPSCs. (**C**) WT mice displayed elevation in the frequency of the sEPSCs 1 h after ketamine (25 mg/kg) injection compared to their saline-injected counterparts, whereas GluN2D KO mice did not (n = 18-21 cells, 5-6 mice/group; ketamine effect, F_1, 78_ = 8.473, *p* = 0.0047; genotype, F_1, 78_ = 5.067, *p* = 0.0272; interaction, F_1, 78_ = 15.79, *p* = 0.0002; WT ketamine vs saline, *p* < 0.0001; GluN2D KO ketamine vs. saline, *p* = 0.7099). (**D**) No significant differences were observed in the amplitude of sEPSC. (**E**) Representative traces of paired-pulse ratio (PPR) at 150 ms inter stimulus interval (**F**, **G**) No significant differences were observed in PPR at any of the inter-stimulus intervals examined (50–200 ms) (n = 13-14 cells, 4-5 mice/group). Electrophysiology data are represented as mean ± SEM. sEPSC, PPR: 2-way ANOVA with Post hoc Sidak’s multiple-comparisons test. **** *p* < 0.0001. *ns*, nonsignificant

WT and homozygous GluN2D KO mice of both sexes were used for experiments, aged 6–16 weeks at the time of recording. Saline or ketamine (25 mg/kg) ((*R*,*S*)-ketamine hydrochloride, Yuhan Corporation, Seoul, Korea) was intraperitoneally administered 1 h before the decapitation. Mice were anesthetized with isoflurane and sacrificed by decapitation in accordance with the regulation and policy approved by Institutional Animal Care and Use Committee in Seoul National University. Coronal slices with 350 μm thickness were obtained as previously described [10].

One or two coronal slices were selected according to their coordinates from Bregma (AP: +1.70) and transferred to a submerged chamber for whole-cell recording, continuously perfused with artificial cerebrospinal fluid (ACSF) that contained (in mM): 124 NaCl, 3 KCl, 26 NaHCO_3_, 1.25 NaH_2_PO_4_, 2 MgSO_4_, 15 d-glucose and 2 CaCl_2_ (carbonated with 95% O_2_ and 5% CO_2_). Pyramidal neurons in layer 5 of the mPFC were recognized by their perpendicular distance from the midline. Recordings were made primarily within the infralimbic cortex, though we could not rule out the possibility that few prelimbic neurons were included. Patch pipettes with a resistance ranging from 1.5 to 6 MΩ were pulled from borosilicate glass and filled with a whole-cell solution comprised (mM): 8 NaCl, 130 CsMeSO_3_, 10 HEPES, 0.5 EGTA, 4 Mg-ATP, 0.3 Na_3_-GTP, 5 QX-314, and 0.1 spermine. The pH was adjusted to 7.2–7.3 with CsOH and osmolarity was set to 290–300 mOsm/l. Neurons were voltage-clamped at − 70 mV throughout the experiment and stabilized at least for 5 min before the recording. Data were accepted for analysis, only if the series resistance values were < 25 MΩ and varied within 20% during the course of the experiment. For sEPSCs experiments, the last 3 min of recording were analyzed using Mini Analysis Program (Synaptosoft Inc., Decatur, GA, USA). The first 25 events from each neuron were used to construct a cumulative histogram.

Ketamine increased the sEPSC frequency of mPFC layer 5 pyramidal neurons 1 h after injection in WT mice, whereas this increase was absent in GluN2D KO mice (Fig. 1B, C). On the other hand, ketamine did not change the sEPSC amplitude of mPFC neurons in either WT or GluN2D KO mice (Fig. 1D). These results may be due to the increase (1) in the number or (2) the presynaptic release probability of functional excitatory synapses onto the layer 5 pyramidal neurons.

To discern these alternative possibilities, we measured paired-pulse ratio, which is known to inversely correlate with presynaptic release probability, at excitatory synapses of layer 5 pyramidal neurons made by inputs from layer 2/3 neurons. The stimulation electrode was placed in layer 2/3 perpendicularly aligned with the patch pipette in layer 5. Two successive electronic simulations were delivered with varying interpulse intervals of 50-200 ms. The paired-pulse ratio was calculated by dividing the peak amplitude of the second EPSC by that of the first EPSC and 4 sweeps were averaged.

Interestingly, no significant differences were observed in the paired-pulse ratio (Fig. 1E–G). Even though we could not exclude the possibility that changes in release probability in other synapses or excitability of presynaptic neurons caused the increase in the sEPSC frequency, PPR data are consistent with the hypothesis that the observed elevation in sEPSC frequency is a result of an increased number of excitatory synapses.

WT mice displayed increased sEPSC frequency of mPFC pyramidal neurons when ketamine was injected (Fig. 1C). Previous research has shown that ketamine predominantly inhibits presynaptic GABAergic interneurons, leading to the disinhibition of pyramidal neurons in the mPFC [3, 11]. However, we consider it unlikely that the elevated sEPSC frequency in the current study was a direct reflection of the disinhibition of excitatory neurons, as ketamine would have been washed out during the preparation of the brain slice. Rather, we speculate that it was a consequence of the synaptic-activity dependent synaptogenesis [12, 13] that might have been induced and stabilized by disinhibition of pyramidal neurons. The resultant increase in the glutamatergic transmission in mPFC likely has augmented dopamine release in both mPFC and striatum resulting in hyperlocomotion, as previously suggested [14, 15].

In contrast, GluN2D KO mice did not show increased sEPSC frequency in response to the ketamine injection. Moreover, they did not develop ketamine-induced hyperlocomotion [5] and the increase of extracellular dopamine level in mPFC in response to phencyclidine, another non-competitive NMDAR antagonist [16]. Given that GluN2D-containing NMDARs are mainly expressed in mPFC interneurons [7], our data suggest a possibility that ketamine disinhibits pyramidal neurons partially by blocking GluN2D-containing NMDARs in interneurons leading to the hyperlocomotion.

In conclusion, the present study adds evidence to the view that GluN2D-containing NMDARs may participate in the process through which ketamine increases glutamatergic synapses of pyramidal neurons in the mPFC, and thereby provides a potential mechanism of ketamine-triggered hyperlocomotion.

## Data Availability

All data in the current study are available from the corresponding author upon reasonable request.

## Abbreviations

NMDAR: *N*-methyl-d-aspartate receptor
mPFC: Medial prefrontal cortex
sEPSC: Spontaneous excitatory postsynaptic currents
WT: Wild-type
KO: Knockout
PPR: Paired-pulse ratio
ACSF: Artificial cerebrospinal fluid
